# Supplementary material for: Thirteen dubious ways to detect conserved structural RNAs
Source: IUBMB Life. Author manuscript; Available in PMC 2024 Jul 10. (PMC11234323; doi:10.1002/iub.2694)
Supplement: supplementary_material [file NIHMS1907110-supplement-supplementary_material.gz › supplemental_material/Table1/CRS.structural.tar.gz!/selected/selected_noRfam/noNUMTs/cmf.M2299369/MyM2299369/R-scape/cmf.M2299369_ext_liftover.hg38.fa_1.R2R.sto.pdf]

cmf.M2299369_ext_liftover.hg38.fa_1

5′-CCUGUCUGGCAGGGGCCGGGGUGCAGGAGGAGGAGACGGCAUCCAGUACAGAGGGGCUGGACUUGGACCCCUGCAGCAGGUACUCGGAGCAAAUGGUGAGAUCAGAAGGGGGAUGAUGUCAUUCCUUCGAAGGAAUGAAUUAAACGUGCUUC
